# Multi-professional primary healthcare centres: psychometric testing of a new quality-of-care instrument

**DOI:** 10.1186/s41687-026-00995-5

**Published:** 2026-01-18

**Authors:** Antoine Dany, Paul Aujoulat, Jean-Yves Le Reste, Delphine Le Goff

**Affiliations:** 1 ER 7479 SPURBO, University of Western Brittany, Brest, France; 2 Department of Human Sciences, University of Western Brittany, Brest, France; 3 Department of General Practice, University of Western Brittany, Brest, France

**Keywords:** Primary health care, Quality of health care, Psychometrics, Multimorbidity

## Abstract

**Background:**

Currently there is no tool to measure the quality of multi-professional primary healthcare centres (MPHCC) that: (i) includes both the patient perspective and the healthcare professional (HCP) perspective, (ii) explores several aspects of care quality, and (iii) has been validated using advanced psychometric methods. However, it is important that MPHCC quality is evaluated by patients with multimorbidity because they are frequent users and require complex and extensive care. Therefore, the purpose of this study was to construct a questionnaire to allow patients with multimorbidity to measure MPHCC quality.

**Methodology:**

An item bank of 72 items structured into nine domains was administered to a large sample of patients with multimorbidity, followed at eight rural or semi-rural MPHCCs. The statistical analyses included methods derived from the Classical Test Theory (Cronbach’s alpha > 0.70) and the Item Response Theory (Loevinger’s H > 0.30) to select items/domains and to ensure robust psychometric properties without being too long for patients.

**Results:**

A total of 507 patients were recruited. The statistical analysis of the participants’ responses allowed developing a questionnaire called QUALSOPRIM, that included only five of the nine original domains: “HCPs’ availability”, “Medical-technical care”, “General practitioner’s expertise”, “Patient-HCP relationships and communication”, and “Main informal caregiver’s role in the care pathway”. Each domain had acceptable internal consistency and met the Item Response Theory assumptions (unidimensionality, monotonicity and local independence).

**Conclusions:**

The QUALSOPRIM questionnaire includes five domains that can be used by patients to assess MPHCC quality. Each domain is succinct and can be used also on its own with relative ease in clinical practice. The psychometric results of the QUALSOPRIM questionnaire must be confirmed in an independent sample of patients to establish its external validity.

**Supplementary Information:**

The online version contains supplementary material available at 10.1186/s41687-026-00995-5.

## Background

Individuals with multimorbidity are the most frequent healthcare service users and the most challenging patients for primary healthcare professionals (HCPs). They often combine many conditions (e.g. chronic diseases, acute diseases, psychosocial and somatic risk factors) and often have complex healthcare situations [1, 2]. The number of such patients is rapidly increasing in all developed countries [3]. To address this issue, healthcare systems are required to coordinate and promote patient-centred, comprehensive care, which is at odds with the classic disease-centred organization [4, 5]. In France, this challenge is partly addressed through the development of multi-professional healthcare centres (MPHCCs) that are promoted, financed and monitored by national health agencies. However, to determine whether these new facilities actually improve patient-centred care, reliable assessments of the user/patient experiences/satisfaction are required. The patient experiences reflect their perceptions of the care they receive and patient satisfaction can give information on the gap between the quality of care received and their expectations [6, 7].

Yet, patient-reported care quality and user experiences are seldom assessed, although their efficacy in encouraging improvement efforts by HCPs has been demonstrated. Moreover, patient-reported outcome and experience measures are appreciated by patients, provided they are not compulsory or excessively time-consuming [8, 9]. Their use is especially important when assessing primary healthcare quality. Indeed, primary care should provide integrated and accessible healthcare services by clinicians who are accountable for addressing the majority of personal healthcare needs, developing a lasting relationship with patients, and also integrating families and the community at large [10].

A previous systematic review showed that many patient self-assessment instruments are available to measure primary care quality [11]. However, a cautious evaluation using the COnsensus-based Standards for the Selection of Health Status Measurement INstruments (COSMIN) checklist [12] found that only one instrument demonstrated that it met Item Response Theory (IRT) requirements (i.e. the Medical Interview Satisfaction Scale, MISS) [13]. The aim of IRT calibration is to produce equal interval scale measures (i.e. a unit of measurement) of a latent trait. A latent trait cannot be directly observed but can be measured by proxies. In the present context, patient-reported quality of care cannot be reliably measured by a single question, but is indirectly measured by items that assess topics correlated with satisfaction. The MISS has 26 items, was originally developed in the United States of America and has excellent psychometric properties. A modified shorter version, MISS-21, was developed in the United Kingdom for use only in general practice but it was never extensively psychometrically assessed [14]. The EUROPEP [15] is a widely used instrument to measure patient satisfaction in general practice developed by an international consortium of researchers and general practitioners (GP). It is available in different languages, but its psychometric properties are modest because it was not developed using IRT. Moreover, no patient self-assessment instrument has been specifically designed to assess complex health facilities such as MPHCCs and/or the challenges associated with multimorbidity.

Therefore, the aim of the QUALSOPRIM project was to develop a valid, responsive, and reliable instrument that can be used by patients to evaluate the quality of primary care, particularly at MPHCCs.

The first step of this project was to generate an item bank from interviews carried out with patients with multimorbidity, informal caregivers and HCPs [16]. This item bank included 109 questions on the patients’ experiences, expectations and satisfaction about their MPHCC medical follow-up. Following this qualitative phase, a pilot study selected items with acceptable face validity [17]. However, the item bank was still too large (*n* = 72 items) to become a usable printed questionnaire and the small patient sample in the pilot study did not allow using advanced statistical analyses (i.e. IRT).

The aim of the present study was to evaluate the 72-item bank in a larger sample of patients with multimorbidity and to use IRT for further reducing the item number and obtaining a domains-structured questionnaire with robust psychometric properties called QUALSOPRIM.

## Methods

### Population

The study was conducted in France in eight rural or semi-rural MPHCCs. No urban MPHCCs was included because this type of healthcare facility was quite new and seldom present in urban areas. Indeed, MPHCCs were initially promoted by the French health authorities to improve access to care in rural and semi-rural areas and they have started to appear in urban areas only recently. The study started in the summer of 2020 and was planned to last one year. Patients were included if they were older than 18 years of age and not subjected to legal restrictions, had at least two chronic conditions and a complex care situation that required at least two HCPs from their MPHCC. Complex situations were defined as an inadequate match between the allocated resources and the encountered health problems. This was objectified by the HCPs’ frustration or by disproportionate costs generated by the misuse of the healthcare system, such as repeated hospitalizations. A purposive sampling method ensured that at least 25% of the included patients had an informal caregiver and at least 25% received medical home visits (a patient could meet both these criteria). As these patients were harder to recruit, all MPHCCs had an inclusion quota to meet. No other criterion was used for sampling. Patient recruitment was performed by one or two GP trainee(s) at each MPHCC. Patients needed to be able to express their consent and provide it in writing. All data were anonymized. The experimental protocol was approved by an ethical research committee on 22 June 2020 (Comités de Protection des Personnes SUD-EST IV) and was categorized as an observational study. At least 500 patients were planned for inclusion, and ~100 items were to be assessed with an item/patient ratio of 1/5 [18].

### Data collection

**Fig. 1:**
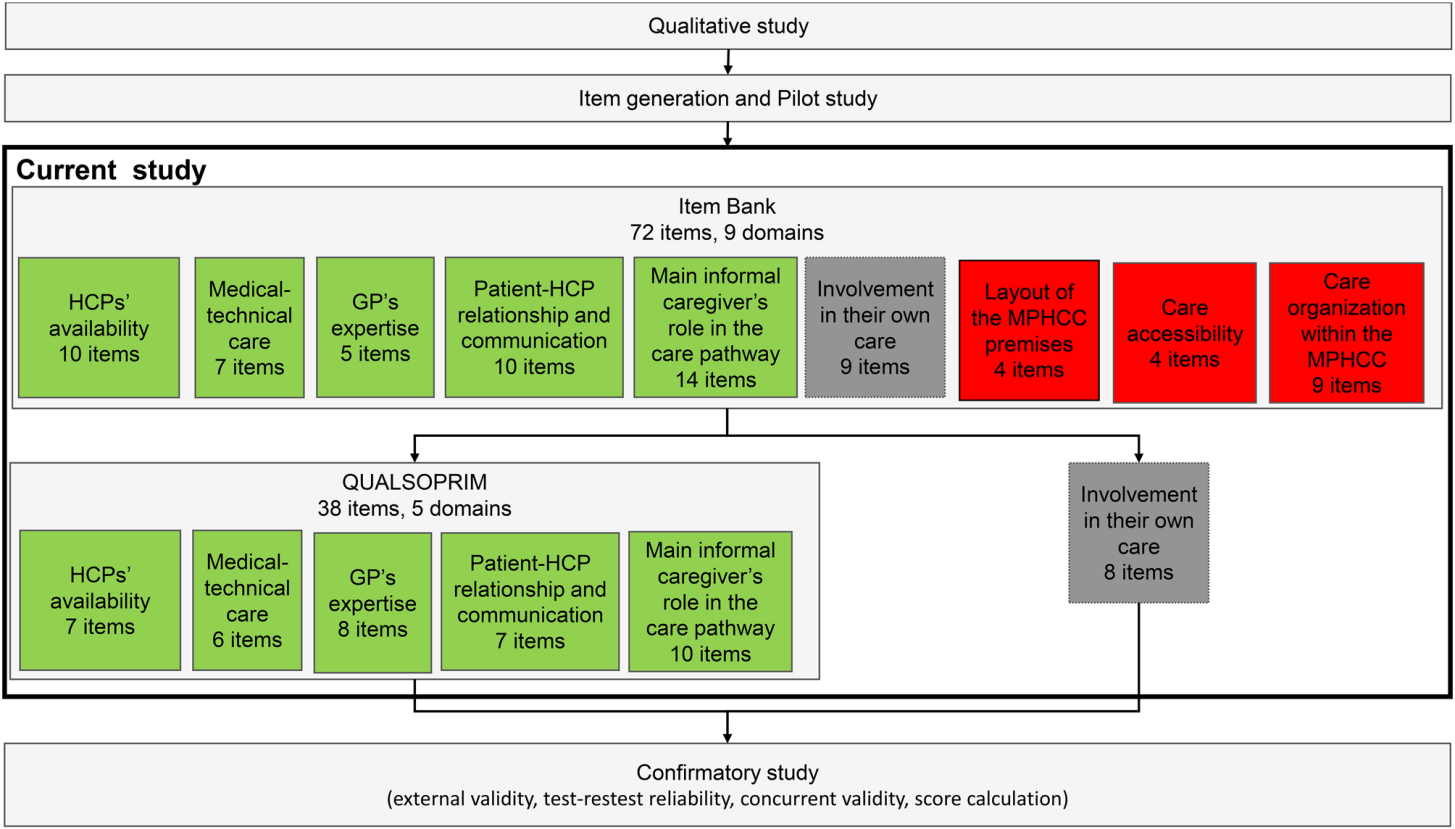
Project flow diagram; MPHCC: multi-professional healthcare centre; HCP: healthcare professional; GP: General practitioner; green: selected domain; red: discarded domain; grey: domain put on hold

Each included patient completed a paper version of the 72-item bank divided in nine domains (see flow diagram in Fig. 1). Each answer was scored using an ordinal scale, a greater score representing a greater satisfaction. In the pilot study [17], neutral response options were added, when necessary, for items for which they were not already available. In the present study, they allowed patients who did not feel concerned or were indifferent towards an item (e.g. “not concerned”, “I do not need etc.”, “I do not know”) to select a proper response option. These neutral responses were treated as intermediate scores (i.e. between the response options expressing unsatisfaction and those expressing satisfaction). This meant that an intermediate score did not reflect the average satisfaction, but neither satisfaction nor unsatisfaction.

### GP trainees’ feedback

GP trainees recruited the patients and explained to the included patients how to fill in the item bank. GP trainees were also asked to report any observation that they deemed relevant. Feedback from GP trainees was considered together with the quantitative results for choosing whether to discard an item or a domain. These would also justify possible small modifications in the questionnaire that will be assessed in the next project phase.

### Mokken scale analysis

The 72-item bank was a priori structured into nine domains on the basis of the previous qualitative and pilot studies (see flow diagram in Fig. 1) [16, 17]. The large dataset collected in the present study was used to assess this a priori structure and adapt it if required. This was achieved using the Mokken scale analysis and the R package ‘Mokken’ [19, 20]. This non-parametric IRT model checks data conformity based on three assumptions: unidimensionality, monotonicity and local independence. In the present study, unidimensionality was respected if all items in a given domain measured a single quality-of-care attribute. Monotonicity was respected if the probability of choosing a response option that reflected better quality of care remained constant or increased as patient satisfaction increased. Local independence implied that the response to one item was not influenced by the responses to other items [21]. These three assumptions are violated if there is a low scalability coefficient (Loevinger’s H). For this coefficient a threshold > 30% was used. The a priori domains with insufficient scalability were discarded. Items from these discarded domains and items that did not fit as anticipated were assessed for inclusion in another domain. Items that did not fit in any domain with acceptable scalability were removed.

### Final construct assessment

The QUALSOPRIM questionnaire will allow patients to assess the different components (i.e. domains) of patient-centred care quality at MPHCCs. Therefore, the scores of the different domains could be used for care quality monitoring within and among MPHCCs and possibly also in other healthcare settings. Therefore, it was important to confirm that each domain reliability assessed only one care quality component.

Once the final structure was established, it was assessed using principal component analysis (PCA) because it is the simplest form of factor analysis (orthogonal angles between axes and no rotation). PCA was performed with the R package ‘ade4’ [22] only to confirm unidimensionality. A parallel analysis (which was performed using the PCA results and the R package ‘nFactors’) allowed determining whether the one-dimension solution was acceptable with a 95% confidence interval [23, 24].

Moreover, the Cronbach’s alpha value was estimated for each domain using the R package ‘psy’ to assess the internal consistency [25]; the acceptability threshold was set at 70% [26]. This statistic was used because it is very popular but it can be misleading. Indeed, it can be artificially improved by increasing the number of items (especially redundant items), and does not require unidimensionality.

### Missing data and statistical analysis tools

No imputation was performed and missing data were handled using listwise deletion for each statistical analysis (i.e. if a missing value prevented a patient from being included in a specific analysis it was removed from it).

All statistical analyses were performed with R programming language version-4.3.2 [27], and the used packages are listed in the relevant analysis.

## Results

### Population

From July 2020 to April 2021, 507 patients were recruited by 14 GP trainees at eight MPHCCs. Their characteristics were typical of patients with multimorbidity: older age, long and frequent follow-up, more women and many chronic conditions: neuro-cardiovascular, musculoskeletal and mental disorders (Table 1, more details in supplementary materials).Patients were recruited during the COVID-19 pandemic; however, the pandemic-related restrictions did not affect recruitment because each GP trainee had a full year to recruit the allocated number of patients (i.e. 36, therefore no amendment to the original protocol was done).

**Table 1:** Patient characteristics. n: number; sd: standard deviation; MPHCCs: multi-professional healthcare centres; HPCs: health-care professionals

Variable	n or mean	% or ±sd
n_total_ = 507 (n reported if < 507)		
**Sex**		
Male	210	41.42
Female	297	58.58
**Age (n = 506)**	73.2	±12
**Follow-up duration (n = 503)**		
< 1 year	15	2.98
1–5 years	172	34.19
5–10 years	126	25.05
> 10 years	190	37.77
**Follow-up visit frequency (n = 500)**		
Several times per month	19	3.8
Once a month	166	33.2
Once every 3 months	286	57.2
Once every 6 months	27	5.4
Once a year or less	2	0.4
**Follow-up visit location**		
Home exclusively	66	13.02
MPHCC exclusively	248	48.92
Both	193	38.07
**Type of non-physician HCP consulted**		
Nurse	370	72.98
Physiotherapist	176	34.71
Podiatrist	126	24.85
Dentist	75	14.79
Others	47	9.27
**Number of conditions**	3.27	1.11
Total conditions reported	1660	100
Diseases of the circulatory system	555	33.43
Sleep-wake disorders	27	1.63
Diseases of the musculoskeletal system or connective tissues	199	11.99
Diseases of the respiratory system	54	3.25
Neoplasms	120	7.23
Endocrine, nutritional or metabolic diseases	336	20.24
Mental, behavioural or neurodevelopmental disorders	66	3.98
Other chronic conditions	131	7.89
Diseases of the nervous system	40	2.41
Diseases of the digestive system	47	2.83
Diseases of the visual system	33	1.99
Diseases of the genitourinary system and Kidney failure	52	3.13

The global results of the present study and their position within the whole project are presented in Fig. 1

### GP trainees’ feedback

As this study was mainly quantitative, feedback was limited, but concerned mainly two points. First, GP trainees almost unanimously reported that patients complained about the questionnaire length. Second, unlike in the pilot study [17], GP trainees rarely reported that some questionnaire items were deemed unimportant or out of the patient’s knowledge.

### Mokken scale analysis

**Fig. 2:**
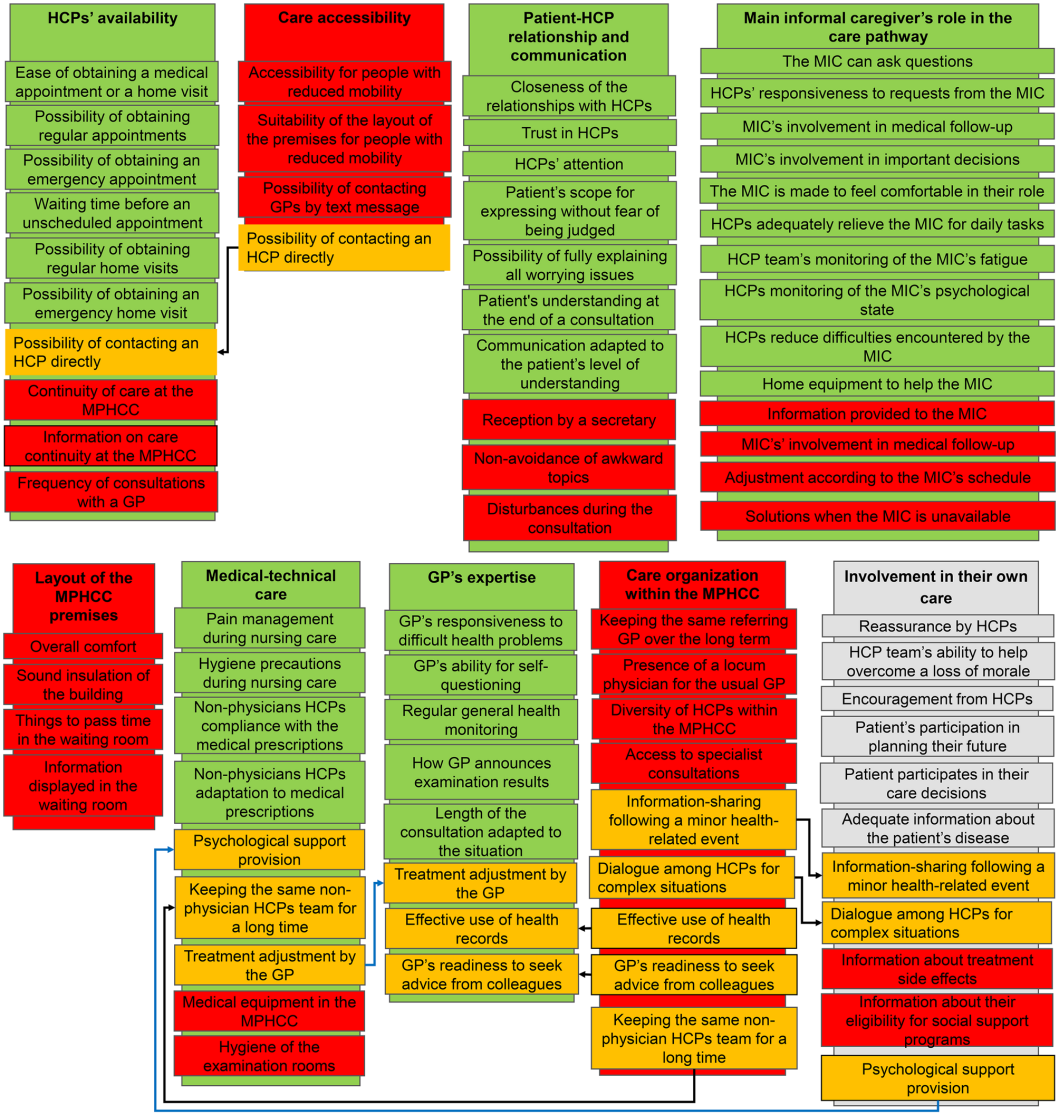
Item and domain selection process; MPHCC: multi-professional healthcare centre; HPC: healthcare professional; GP: General practitioner; MIC: main informal caregiver; green: selected item/domain; orange: reclassified item; red discarded item/domain; grey: item/domain put on hold; black arrow: item reclassification from a discarded domain; blue arrow: item reclassification from a selected domain

Of the nine a priori domains (Fig. 1), five were retained, three were discarded, and one was kept for further evaluation. The amendments made to the a priori structure to form the definitive structure of the self-administered QUALSOPRIM questionnaire are summarized in Fig. 2.

Three domains were removed: Care accessibility, Layout of the MPHCC premises, and Care organization within the MPHCC. Specifically, the Care accessibility domain was discarded because no scalability-based association of more than two items was found. Only one of the four items (“Possibility of contacting an HCP directly”) had a scalability coefficient > 30% in the “HCPs’ availability” domain and therefore, was added to this domain.

All items in the Layout of the MPHCC premises domain were removed because they did not form a domain and none found its place in another domain on the basis of the Mokken scale analysis results.

The Care organization within the MPHCC domain did not meet the IRT requirements and was removed. However, five of the nine items were added to other domains. The “Keeping the same non-physician HCPs for a long time” item was added to the “Medical-technical care” domain. The “Effective use of health records” and “GPs readiness to seek advice from colleagues” items were added to the “GPs expertise” domain. The last two items, namely, “Dialogue among HCPs for complex situations” and “Information-sharing among HCPs following minor health problems” were added to the “Involvement in their own care” domain.

### Final construct assessment

Of the six retained domains and after all item reclassifications, five met the IRT requirements (Fig. 1). The domains and items that were retained are presented in Table 2 with their psychometric properties.

**Table 2:** Domain composition and psychometric properties. Numbers in bold indicate metrics below the corresponding acceptable threshold

	Item’s H	Domain’s metrics	
**HCPs’ availability**			
1. Ease of obtaining a medical appointment or a home visit	0.41		n = 473
2. Possibility of obtaining regular appointments	0.4		H = 0.36
3. Possibility of obtaining an emergency appointment	0.37		Cronbach ɑ = 0.70
4. Waiting time before an unscheduled appointment	**0.29**		**PA = 2**
5. Possibility of obtaining regular home visits	0.38		
6. Possibility of obtaining an emergency home visit	0.35		
7.Possibility of contacting an HCP directly	0.35		
**Medical-technical care**			
1. Pain management during nursing care	0.41		n = 482
2. Hygiene precautions during nursing care	0.39		H = 0.37
3. Non-physician HCPs’ compliance with the medical prescriptions	0.4		Cronbach ɑ = 0.73
4. Non-physician HCPs’ adaptation to medical prescriptions	0.43		PA = 1
5. Keeping the same team of non-physician HCPs	**0.27**		
6. Psychological support provision	**0.25**		
**General practitioner’s ** **expertise**			
1. GPs responsiveness to difficult health problems	0.43		n = 464
2. GPs ability for self-questioning	**0.29**		H = 0.35
3. Regular general health monitoring	0.41		Cronbach ɑ = 0.71
4.How GP announces examination results	0.35		PA = 1
5. Length of the consultation adapted to the situation	0.32		
6. Treatment adjustment by the GP	0.4		
7. Effective use of health records	**0.29**		
8. GPs readiness to seek advice from colleagues	0.35		
**Patient-HCP relationship and communication**			
1. Closeness of the relationships with HCPs	0.45		n = 465
2. Trust in HCPs	0.44		H = 0.40
3. HCPs’ attention	0.55		Cronbach ɑ = 0.78
4. Patient’s scope for expressing without fear of being judged	0.41		PA = 1
5. Possibility of fully explaining all worrying issues	0.33		
6. Patient’s understanding at the end of a consultation	0.32		
7. Communication adapted to the patient’s level of understanding	**0.28**		
**Involvement in their own care**			
1. Reassurance by HCPs	0.33		n = 422
2. HCP team’s ability to help overcome a loss of morale	0.3		H=**0.28**
3. Encouragement from HCPs	0.31		Cronbach ɑ=**0.68**
4. Patient’s participation in planning their future	**0.23**		**PA = 2**
5. Patient participates in their care decisions	0.3		
6. Adequate information about the patient’s disease	0.31		
7. Dialogue among HCPs for complex situations	**0.22**		
8. Information-sharing following a minor health-related event	**0.26**		
**Main informal caregiver’s role in the care pathway**			
1. The informal caregiver can ask questions	0.4		n = 74
2. HCPs’ responsiveness to requests from the informal caregiver	0.6		H = 0.44
3. Informal caregiver’s involvement in medical follow-up	0.37		Cronbach ɑ = 0.83
4. Informal caregiver’s involvement in important decisions	0.37		PA = 1
5. The informal caregiver is made to feel comfortable in their role	0.41		
6. HCPs adequately relieve the informal caregiver for daily tasks	0.43		
7. HCP team’s monitoring of the informal caregiver’s fatigue	0.52		
8. HCPs monitoring of the informal caregiver’s psychological state	0.5		
9. HCPs reduce difficulties encountered by the informal caregiver	0.52		
10. Home equipment to help the informal caregiver	0.37		

MPHCCs: multi-professional healthcare centres; HCPs: health care professionals; GP: General practitioner; n: number of patients on which the analyses were performed; H: Loevinger’s coefficients; PA: parallel analysis

HCPs’ availability - Six items were retained from the a priori classification and one item (Possibility of contacting a HCP directly) was moved from the “Care accessibility” domain. In all, this domain contained seven items. The PCA found two dimensions instead of one, invalidating that the items measured a single underlying concept.

Medical-technical care - Four items were retained from the a priori classification and two items were added following reclassification: “Keeping the same non-physician HCPs team for a long time” from the discarded “Care organization within the MPHCC” domain, and the other item concerned psychological support provision from the “Involvement in their own care” domain. The domain included six items. The PCA confirmed that the items measured a single underlying concept.

General practitioner’s expertise - All five items were retained from the a priori classification and three items were added following reclassification: the “Treatment adjustment by the GP” item from the “Medical-technical care” domain, and the “Effective use of health records” and “GPs readiness to seek advice from colleagues” items from the discarded “Care organization within the MPHCC” domain. In all, the domain included eight items. The PCA confirmed that the items measured a single underlying concept.

Patient-HCP relationships and communication - Seven items from the a priori classification were retained. The PCA confirmed that the items measured a single underlying concept.

Involvement in their own care - Six of nine items from the a priori classification were retained, and two were added after reclassification: “Dialogue among HCPs for complex situations” and “Information-sharing following minor health problems” from the discarded “Care organization within the MPHCC” domain. In all, the domain included eight items. The PCA found two dimensions instead of one, invalidating that the items measured a single underlying concept.

Main informal caregiver’s role in the care pathway - Ten items were retained from the a priori classification. In all, the domain included ten items. The PCA confirmed that the items measured a single underlying concept.

A culturally non-validated translation in English of the QUALSOPRIM questionnaire is available in Supplementary material.

## Discussion

The self-administered QUALSOPRIM questionnaire is the first instrument to measure MPHCC quality of care, as assessed by patients with multimorbidity. It was developed to meet the IRT requirements and using both patient and professional feedback. It is composed of 41 items, 3 general items and, 38 items in one of the five specific domains. These results are promising but need to be confirmed in another sample to prove the external validity. The domains of the QUALSOPRIM questionnaire are independent and can be used separately.

The “Involvement in their own care” domain barely failed the IRT assessment. It could have been discarded, but we think that we can improve it, because patients’ involvement in their care is often promoted in the current care management [28]. It is also identified as a core aspect of care for patients with multimorbidity who often experience complex situations, where their involvement could improve relationships with HCPs, their morale, and the relevance of important decisions, and help them adopt healthier behaviours [29, 30]. As this domain is a critical element of patient-centred care and it was very close to meeting the IRT requirements, we decided to retain it for the last phase of the study.

The “HCPs’ availability” domain was considered validated, despite failing its unidimensionality assessment. This lack of fit may stem from the inclusion of both urgent and non-urgent availability items. Given that the violation of unidimensionality was minor, it was deemed acceptable for the current stage of development. However, should this issue persist in the next phase, the domain may need to be excluded.

The QUALSOPRIM questionnaire was simplified and structured using IRT. It is now shorter and adaptable. Therefore, it stands a good chance of being useful in busy clinical settings for the quality assessment of MPHCCs and in primary care research. It could also be used to provide reliable subjective metrics to enable the implementation of evidence-based quality monitoring policies for MPHCCs.

The five final domains concern topics that are currently considered essential for primary care quality [31]. Conversely, the discarded domains presented two major limitations. First, neither MPHCC accessibility nor the layout of the premises were an issue for most patients. Second, the Care organization within the MPHCC domain was not easy for the patients to understand. This supported our patient perspective-based approach. These three topics, which are considered relevant by HCPs and health regulation agencies, turned out to be unimportant or too difficult to assess by most patients in our study. For example, most patients were not bothered by their MPHCC layout or accessibility, unless it was really uncomfortable or difficult to reach. They were often caught off guard when asked about care organization and thought they were in no position to evaluate these topics. Therefore, we think that it may be more relevant to leave such assessments to professionals.

The present study took place in a single country and cross-cultural validation would be necessary before the QUALSOPRIM questionnaire can be used in another country. No urban MPHCC was included and this may also limit our results generalizability. A future study in this type of MPHCC could be valuable. However, as semi-rural MPHCCs also attract a significant part of urban population, the effect should be limited. The present study was hampered by the time required to cover the whole item bank. Thus, patients had to remain focused and interested in providing responses. Although the patients were informed and received encouragement from the GP trainees, the responses to items in the last two domains could have been affected by respondent fatigue. Although we did not find any quantitative impact (e.g. excess of missing data) in these domains, the GP trainees often reported that patients complained about the questionnaire length. These domains were “Involvement in their own care”, which was retained despite its low compatibility with the IRT requirements, and “Main informal caregiver’s role in the care pathway”, which met the IRT requirements. The present study did not evaluate the concurrent validity and the test-retest validity because the participation time required for each patient was already important. This is planned for the next phase of the project (see flowchart in Fig. 1).

Patients with multimorbidity form a very heterogeneous group with very different needs. Their most notable common feature is that they require lengthy consultations with competing treatment priorities in long-term care [32]. No single-format questionnaire can satisfactorily assess the profiles of such heterogenous sample of patients. However, the domains’ independence and neutral-response options of the QUALSOPRIM questionnaire allow relevant items/domains to meet the specific patient profiles. This can be useful for example if one wants to use only one specific domain in a specific context or for specific stakeholders (e.g. focusing on HCPs’ availability or caregiver involvement).

Compared with MISS-21 [14] or EUROPEP [15, 33], the QUALSOPRIM questionnaire was specifically developed for patients with multimorbidity in complex primary care health facilities (i.e. MPHCCs). It was developed from the start to meet the IRT requirements. Its usability remains to be proved outside a research protocol, but it is longer than the other two questionnaires (41 items vs 21 and 23, respectively). Despite these differences, comparing the EUROPEP instrument and the QUALSOPRIM questionnaire would have been interesting to assess the concurrent validity because they have similarities. Indeed, the EUROPEP instrument was validated to assess general practice care quality by various kind of patients, in various kind of settings and in different countries. However, this was not feasible in the present study because it would have further increased the participation time required for each patient, which was already substantial.

## Conclusion

The QUALSOPRIM domains are scaled to produce reliable measures for MPHCC quality assessment by patients with multimorbidity. The questionnaire mean scores could be used to monitor changes in a single MPHCC or to compare several MPHCCs. To finalize the project, and before routine use, the compatibility with the IRT requirements of each domain will need to be confirmed in another sample [34]. Furthermore, both test-retest reliability and concurrent validity will be assessed (using the EUROPEP instrument).

## Electronic supplementary material

Below is the link to the electronic supplementary material.

Supplementary Material 1

## Data Availability

The datasets used and/or analysed during the current study are available from the corresponding author on reasonable request.

## Abbreviations

GP: General practitioner
HCP: Health care professional
IRT: Item Response Theory
MPHCC: Multi-professional healthcare centre
