# Supplementary material for: Multi-professional primary healthcare centres: psychometric testing of a new quality-of-care instrument
Source: J Patient Rep Outcomes. 2026 Jan 18;10:21. doi: 10.1186/s41687-026-00995-5 (PMC12894520; doi:10.1186/s41687-026-00995-5)
Supplement: Supplementary file 1 — Supplementary Material 1 [file 41687_2026_995_MOESM1_ESM.docx]

# Patients’ characteristics

**Condition types as defined by the international classification of diseases 11^th^ version (ICD-11):**

Diseases of the circulatory system including essential hypertension.

Diseases of the musculoskeletal system or connective tissues including gout.

Endocrine, nutritional or metabolic diseases including disorders of the thyroid gland or thyroid hormones system, diabetes mellitus, hypercholesterolaemia, and obesity.

Other chronic conditions include complex regional pain syndrome, chronic pain, oesophagitis, chronic widespread pain, laryngeal spasm, lupus erythematosus, factors influencing health status or contact with health services, diseases of the blood or blood-forming organs, diseases of the skin, diseases of the immune system, developmental anomalies, certain infectious or parasitic diseases, chronic neuropathic pain, vertigo, and diseases of the ear or mastoid process.

# Questionnaire

**QUALSOPRIM**

**Date: Place: Patient number:**

# Availability of healthcare professionals

- 1. **Can you easily obtain an appointment for a visit? (at the healthcare centre or home visit)**

easily

4

quite easily

3

not very easily

2

with difficulty

1

- 1. **How would you rate the waiting time to obtain an appointment for a non-urgent visit?**

reasonable

4

quite reasonable

3

rather unreasonable

2

unreasonable

1

- 1. **How would you rate the waiting time to obtain an appointment for an urgent visit? (acute health problem)**

reasonable

3

quite reasonable

2

1

unreasonable

not concerned

0

- 1. **How would you rate the waiting time for a walk-in visit?**

satisfactory

2

unsatisfactory

1

not concerned

0

- 1. **How would you rate the waiting time to obtain an appointment for a non-urgent home visit?**

reasonable

3

quite reasonable unreasonable

2

1

not concerned

0

- 1. **How would you rate the waiting time to obtain an appointment for an urgent home visit? (acute health problem)?**

reasonable

2

unreasonable

1

not concerned

0

- 1. **When you wish to talk to a healthcare professional, can you reach them directly?**

easily

3

quite easily

2

1

with difficulty

not concerned

0

# Medical-technical care

- 1. **Do nurses take enough precautions to limit pain during your care?**

they are very attentive

2

sometimes they do not pay attention

1

I do not need any nursing care

0

- 1. **Do nurses follow sufficiently the hygiene standards during your care?**

they always pay attention

2

sometimes they do not pay attention

1

I do not need any nursing care

0

- 1. **Are you satisfied with the non-physician HCP staff's compliance with medical prescriptions?**

fully satisfied

2

not always satisfied

1

I do not need non-physician care

0

- 1. **Are you satisfied with the non-physician HCP staffs’ ability to adapt to changes in prescriptions? (in function of how your health status changes; for instance, dressing adapted to the different stages of a wound)**

fully satisfied

2

not always satisfied

1

I do not need non-physician care

0

- 1. **For your regular care, are you always followed by the same team of healthcare professionals (non-physician)?**

most of the time

2

not often enough

1

I do not need non-physician care

0

- 1. **Are your satisfied with the proposed psychological support?**

fully satisfied

2

not always satisfied

1

I do not feel concerned

0

# General practitioner’s expertise

- 1. **Is your doctor reactive enough when you have health problems for which it is difficult to make a diagnosis? (e.g. prescription of additional tests, referral to specialists)**

reactive

4

quite reactive

3

not very reactive

2

very unreactive

1

- 1. **Does your doctor accept to reassess/call into question a diagnosis when you consult several times for the same health problem without any improvement?**

my doctor is willing to reassess the diagnosis or treatment strategy

2

I have to insist on being heard and get my doctor to question the previous decisions

1

not concerned

0

- 1. **Are you satisfied with the regularity of your health follow-up in general? (e.g. screening, blood tests, changes in your chronic disease)**

my follow-up is regular and of good quality

2

my follow-up is sometimes irregular

1

- 1. **Are you satisfied with how your doctor explains to you the results of your exams? (e.g. blood tests, X-ray exams)**

very satisfied

3

satisfied

2

sometimes unsatisfied

1

- 1. **Does your doctor sufficiently adapt the consultation time in function of the health problem that led you to book the visit?**

flexible within reason

3

fairly/quite flexible

2

not very flexible

1

- 1. **Does your doctor adapt your treatment as your disease progresses? (e.g. modification of the treatment/dose, home help)**

as soon as required

2

my doctor is sometimes slow in doing it

1

- 1. **When you see doctors, do you think that they make effective use of your health record? (e.g. your history, allergies)**

most of the time

2

not sufficiently

1

- 1. **Do you think that your doctor will easily contact a colleague to ask for an opinion in the event of doubts about your care?**

readily seeks advice if in doubt

2

sometimes delays asking a colleague for advice

1

not concerned

0

# Patient-healthcare professional relationships and communication

- 1. **Do you think that communication with the healthcare professionals at the healthcare centre is adapted to your level of understanding?**

perfectly adapted

2

sometimes not adapted

1

- 1. **Are you satisfied with the relational proximity that you can have with your healthcare professionals?**

satisfied, they know how to set the right distance

4

quite satisfied

3

rather unsatisfied

2

unsatisfied

1

- 1. **Do the healthcare professionals inspire trust/confidence?**

I feel fully trustful

4

I feel quite trustful

3

I'm rather distrustful

2

I do not trust them

1

- 1. **During consultations, do you think that the healthcare professionals are listening to you?**

fully

4

quite well

3

rather insufficiently

2

insufficiently

1

- 1. **Do you feel free to express yourself without fear of remarks from the healthcare professionals?**

fully free

4

quite free

3

rather holding back

2

really holding back

1

- 1. **Have you ever left a consultation without having been able to clearly explain what was worrying you?**

never or rarely

4

sometimes

3

often

2

most of the time

1

- 1. **Did it happen already that you left a consultation without understanding what the healthcare professional(s) had told you?**

never or very rarely

2

yes

1

# Involvement in their own care

- 1. **Have you been adequately informed about your disease? (e.g. course, prognosis, possible treatments, physical consequences)**

I am very well informed

3

I am reasonably well informed

2

I am not very well informed

1

- 1. **Do healthcare professionals reassure you throughout your disease?**

reassuring, they always have a comforting word to say

3

fairly reassuring

2

not very reassuring

1

- 1. **When you are feeling low, are you satisfied with how the healthcare team helps you to get through the ordeal? (e.g. medications, moral support)**

satisfied

3

quite satisfied

2

rather unsatisfied

1

not concerned

0

- 1. **Do healthcare professionals encourage you throughout your disease? (e.g. diet, following your treatment)**

most of the time

2

sometimes

1

- 1. **Do healthcare professionals allow you to play a sufficiently active role in planning your future? (e.g. staying at home, moving to a retirement home)**

I feel fully involved

2

I feel little or only moderately involved

1

not concerned

0

- 1. **Do you feel that you are in charge of your care and do you make decisions about your health together with the healthcare professionals?**

I feel fully involved

2

I feel little or moderately involved

1

- 1. **Do the healthcare professionals consult each other when dealing with complex situations? (e.g. clinical, social, etc.)**

they formally meet for decision making

2

they work independently or exchange ideas informally

1

I do not know

0

- 1. **In the event of a minor event concerning your health, do healthcare professionals share rapidly the required information?**

rapidly

3

quite rapidly

2

rather slowly

1

I do not know

0

# Main informal caregiver’s role in the care trajectory

- 1. **Do you have an informal caregiver: a person who helps you in your daily life to cope with your health condition?**

yes

1

0 no ⇒ go to section G (page 12)

- 1. **Do healthcare professionals ensure that your informal caregiver can ask questions?**

most of the time

2

not always

1

- 1. **Are the healthcare professionals at the healthcare centre receptive to your informal caregiver’s requests? (e.g. reachable by the carer)**

receptive

2

rather resistant or not always receptive

1

- 1. **Do healthcare professionals involve your informal caregiver in understanding your disease and its management?**

most of the time

2

not always

1

- 1. **Do healthcare professionals involve your informal caregiver in planning your future? (e.g. end of life, moving to an adapted structure)**

my caregiver is always involved in the decisions concerning my future

2

not always

1

- 1. **Do you think that the healthcare professionals make your informal caregiver feel comfortable in their role?**

most of the time

2

not always

1

- 1. **Concerning the daily tasks related to your health condition, does the healthcare professional team relieve sufficiently your informal caregiver?**

most of the time

2

not always

1

- 1. **Do healthcare professionals monitor your informal caregiver’s fatigue level?**

most of the time

2

not always

1

- 1. **Do healthcare professionals monitor your informal caregiver’s psychological state?**

most of the time

2

not always

1

- 1. **Do healthcare professionals try to reduce the difficulties that your informal caregiver encounters at home? (e.g. adapting the house, proposing help)**

most of the time

2

not always

1

- 1. **Does the equipment in your home facilitates your informal caregiver’s daily life? (e.g. patient lifter, nursing bed, commode chair)**

very much

2

little or moderately

1

# Overall satisfaction

- 1. **Today, do you think that your health has improved or that you are living well despite your health problems? (e.g. constraints, pain, regular care)**

I feel well, stabilised, and less in pain

4

I feel quite well, stabilised, and a little less in pain

3

my disease is always a burden, but I feel supported and well cared for

2

I am not satisfied with my health state and my disease course

1

- 1. **Are you satisfied with your care at the healthcare centre?**

satisfied

3

quite satisfied

2

rather unsatisfied

1

- 1. **Are you satisfied with the healthcare teams intervening at home?**

satisfied

2

little or moderately satisfied

1

not concerned

0
